# Practices and Obstacles to Provider-Initiated HIV Testing and Counseling (PITC) Among Healthcare Providers in Côte d’Ivoire

**DOI:** 10.1007/s10461-020-02923-0

**Published:** 2020-05-24

**Authors:** Maxime Inghels, Arsène Kra Kouassi, Serge Niangoran, Anne Bekelynck, Séverine Carillon, Lazare Sika, Christine Danel, Mariatou Kone, Annabel Desgrees du Lou, Joseph Larmarange, for the ANRS 12323 DOD-CI Study Group, Nelly Assoumou, Mohamed Doumbia, Alexis Kouadio, Honoré Ouantchi

**Affiliations:** 1 Lincoln International Institute for Rural Health (LIIRH), University of Lincoln, Brayford Pool, Lincoln, Lincolnshire LN6 7TS UK; 2 Centre Population et Développement (UMR 196 Paris Descartes – IRD), SageSud (ERL INSERM 1244), Institut de Recherche Pour Le Développement, Paris, France; 3 Programme PAC-CI/ANRS, Centre Hospitalier Universitaire de Treichville, Abidjan, Ivory Coast; 4 École Nationale Supérieure de Statistique et d’Economie Appliquée (ENSEA), Abidjan, Ivory Coast; 5 Centre Inserm 1219, Université de Bordeaux, Bordeaux, France; 6 Institut D’Ethno-Sociologie (IES), Abidjan, Ivory Coast

**Keywords:** Provider initiated testing and counselling, HIV testing, Côte d’Ivoire, Health personnel, Phone-based survey

## Abstract

Practices of Provider-Initiated HIV Testing and Counseling (PITC) remains suboptimal in Côte d’Ivoire. *The aim of this survey was *to identify the practices and obstacles to PITC among healthcare professionals in Côte d'Ivoire. A nationally representative cross-sectional survey was conducted in 2018 by telephone among three separate samples of midwives, nurses and physicians practicing in Côte d'Ivoire. The number of HIV tests proposed during consultation in the month preceding the survey was collected for each professional. Factors associated with the number of proposed tests were identified through ordinal logistic regression models. A total of 298 midwives, 308 nurses and 289 physicians were interviewed. Midwives proposed the test more frequently, followed by nurses and physicians. Among midwives, a higher number of proposed tests was associated with the perception that HIV testing does not require specific consent compared to other diseases (aOR 4.00 [95% CI 1.37–14.29]). Among nurses, having received HIV training and the presence of community HIV counselors were associated with a higher number of proposed tests (aOR 2.01 [1.31–3.09] and aOR 1.75 [1.14–2.70], respectively). For physicians, the presence of a voluntary testing center was associated with a higher number of proposed tests (aOR 1.69 [1.01–2.86]). PITC practices and barriers differed across professions. Beyond improving environmental opportunities such as dedicated staff or services, strengthening the motivations and capabilities of healthcare professionals to propose testing could improve PITC coverage.

**Electronic supplementary material:**

The online version of this article (10.1007/s10461-020-02923-0) contains supplementary material, which is available to authorized users.

## Resumen

Las prácticas de Asesoramiento y Pruebas de VIH Iniciadas por el Proveedor (APIP) siguen siendo subóptimas en Côte d'Ivoire. El objetivo de este estudio fue identificar las prácticas y los obstáculos a las APIP entre los profesionales de la salud en Côte d'Ivoire. En 2018 se llevó a cabo una encuesta transversal representativa a nivel nacional por teléfono entre tres muestras separadas de parteras, enfermeras y médicos que ejercen en Côte d'Ivoire. Se recogió para cada profesional el número de pruebas de VIH propuestas durante la consulta en el mes anterior a la encuesta. Los factores asociados con el número de pruebas propuestas se identificaron mediante modelos ordinales de regresión logística. Se entrevistó a un total de 298 parteras, 308 enfermeras y 289 médicos. Las parteras propusieron la prueba con mayor frecuencia, seguidas de las enfermeras y los médicos. Entre las parteras, un mayor número de pruebas propuestas se asoció a la percepción de que la prueba del VIH no requiere un consentimiento específico en comparación con otras enfermedades (aOR 4,00 [IC del 95%: 1,37-14,29]). Entre las enfermeras, el haber recibido capacitación sobre el VIH y la presencia de consejeros comunitarios sobre el VIH se asociaron con un mayor número de pruebas propuestas (aOR 2,01 [1,31-3,09] y aOR 1,75 [1,14-2,70], respectivamente). Para los médicos, la presencia de un centro de pruebas voluntarias se asoció con un mayor número de pruebas propuestas (aOR 1,69 [1,01-2,86]). Las prácticas y barreras del APIP difieren entre las profesiones. Más allá de mejorar las oportunidades del entorno, como el personal o los servicios dedicados, el fortalecimiento de las motivaciones y capacidades de los profesionales de la salud para proponer pruebas podría mejorar la cobertura del APIP.

## Background

Because of the limitations of voluntary HIV testing (patient-initiated testing) and the insufficient coverage of HIV testing, the World Health Organization has recommended Provider-Initiated HIV Testing and Counseling (PITC) since 2007 [1]. PITC adoption in the majority of sub-Saharan countries has contributed significantly to the increase in HIV testing coverage [2–4]. Despite its systematic recommendation to certain audiences (e.g., pregnant women) or in certain epidemiological contexts (i.e., areas with HIV prevalence above 1%), routine PITC implementation has remained suboptimal in sub-Saharan Africa [5]. Test proposal rates in recommended situations by a healthcare professional vary between 24 and 94% across studies [5].

Some studies have highlighted barriers faced by healthcare professionals in their PITC practice. At the individual level, poor perception of the PITC [6], lack of motivation [7–10] and difficulties in offering testing or counseling [8–12] are mentioned, as are the workload associated with PITC [8, 11, 13] and the lack of specific training [9, 11, 14–17]. At the structural level, a lack of trained staff [7, 8, 12, 14], shortages of testing kits [10, 11, 13, 16] and inadequate space that does not guarantee confidentiality [7–14] are associated with low PITC practice. Some studies have suggested that PITC organization (i.e., professionals performing the different steps of testing, patient referrals or no referrals for testing) can facilitate test proposal [15, 18, 19].

Since 2009, routine PITC has been recommended in Côte d’Ivoire to all patients, regardless of their reason for consultation [20]. Nevertheless, the proposal rates observed in consultation remain low. According to 2017 national data, only 15.2% of 12,955,898 consultations conducted nationally were documented with a test proposal, and 75.9% of these proposals resulted in HIV testing [21]. These figures were consistent with another study that also found a test proposal rate of approximately 20% in outpatient services [22]. In a country where the HIV prevalence was 2.8% in 2017 and where only 54% of people living with HIV knew their status, it crucial to understand, from the perspective of healthcare professionals, what factors influence testing proposal practices [23, 24].

To our knowledge, no studies have documented the obstacles to the practice of PITC at the national level in Côte d'Ivoire. The objective of this analysis is therefore to describe PITC practices by healthcare professionals and to identify factors associated with this practice.

## Methods

A cross-sectional survey was conducted between February and November 2018 by telephone among midwives, nurses and physicians throughout Côte d'Ivoire. Lists of telephone contacts were obtained from the Ministry of Health for nurses and midwives and from the Ivorian Medical Association for physicians. A random sample (equal probabilities) was selected from each list. In the case of phone contact, the survey was presented, and informed consent was orally obtained. In the absence of contact, each telephone number was dialed up to 15 times consecutively without contact before being considered unreachable.

**Fig. 1:**
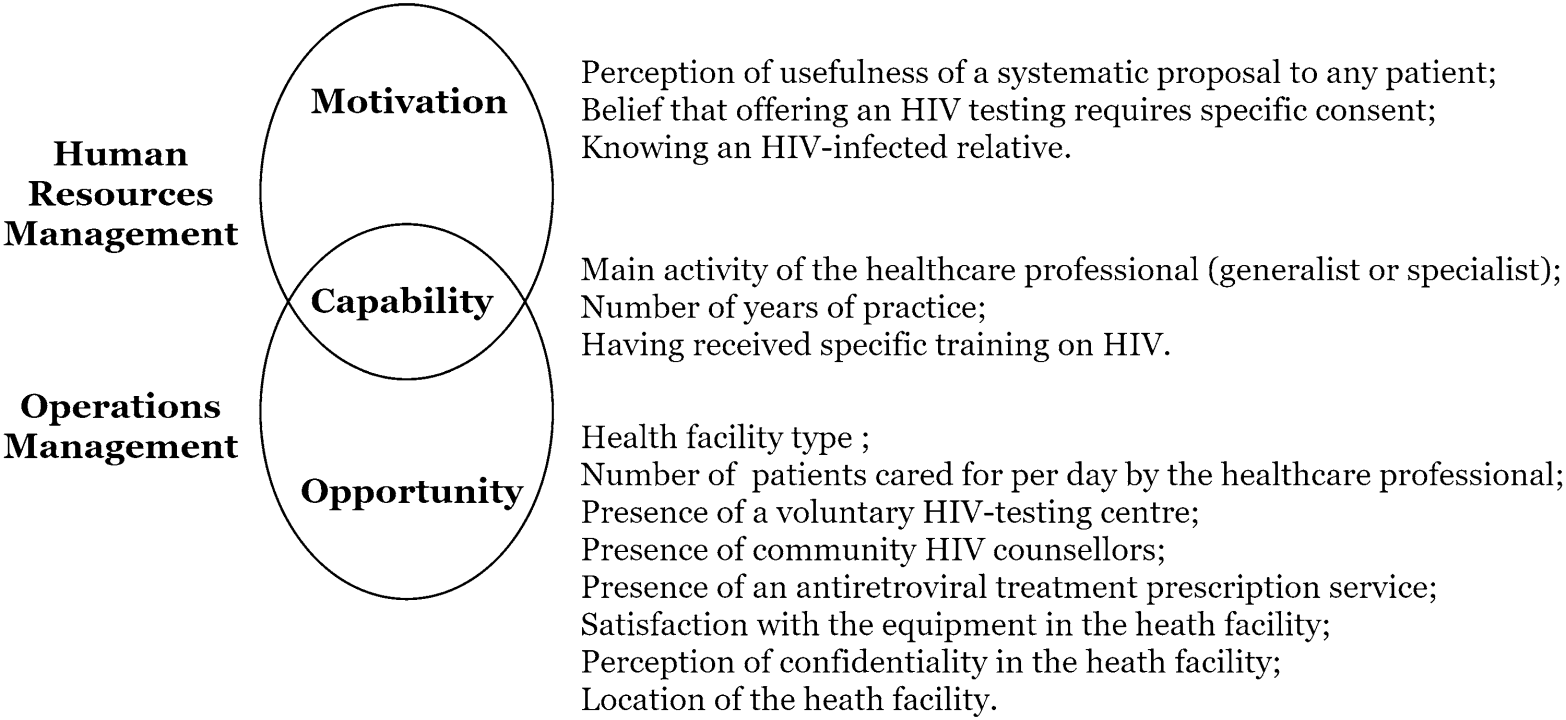
Measured factors with practice and obstacles to provider-initiated HIV testing and counseling according to the Motivation-Opportunity-Capability framework, adapted from Boudreau et al. [24]

Healthcare professionals were asked about the number of HIV tests they proposed in consultation during the previous month (none, between 1 and 5, between 6 and 10, between 11 and 20, and 21 or more). Other collected data concerned healthcare professionals’ characteristics, their professional training and the characteristics of their main facility (i.e., the one in which they practiced most often). Potential factors were classified according to three dimensions directly inspired by the conceptual model of work performance [25]: motivation, capability and opportunity (Fig. 1).

According to this model, motivation is defined as the desire and willingness of professionals to act. In our survey, motivation included the perceived usefulness of a systematic proposal of an HIV test to any patient, the belief that offering an HIV test requires specific consent compared to other tests and a relative living with HIV.

Capabilities are defined as the skills, knowledge and abilities necessary to carry out an action in line with the objectives of the health structure. This concept can be applied both at the level of the healthcare professional and at the level of his/her facility. In our survey, we measured the main activity of the healthcare professional (general practitioner or specialist, with all midwives considered specialists), the number of years of practice and having received specific training on HIV.

Opportunity refers to situations encountered within the institution that facilitate the execution of actions and their expected effects. In our survey, opportunity included the type of health facility (hospital, health center, private or other) and, within this facility, the number of patients seen per day by the surveyed healthcare professional and the presence of a voluntary HIV testing center, community HIV counselors, and/or an antiretroviral treatment (ARV) prescription service. In addition, satisfaction with the available equipment, the perception of confidentiality in the facility and its location (Abidjan, urban or rural departments) were collected.

The analyses were stratified by profession (midwife, nurse and physician). Bivariate analyses were conducted to describe associations with the number of proposed tests in the month preceding the survey. Fisher’s exact tests were used to calculate the corresponding p-values. The significant variables at the 0.20 threshold in the bivariate analyses were included in multivariate ordinal logistic regression models. The ordinal categories of the number of proposed tests in the preceding month were none, between 1 and 5, between 6 and 10, between 11 and 20, and 21 or more. A step-by-step downward selection using the Akaike information criterion was performed to obtain the final models. Likelihood ratio tests were used to calculate the global p-values in these models. We used R (version 3.3.5) and the *ordinal* package [26].

## Results

### Sample description

Overall, 1450 telephone numbers were called (425 midwives, 425 nurses, 600 physicians). After excluding off-target numbers, 1329 healthcare professionals were eligible for the survey. The participation rates (taking into account refusal rates and execution failures) were 75.8%, 83.3% and 58.3% for midwives, nurses and physicians, respectively (Table S1, Supplemental Digital Content—SDC).

A total of 299 midwives, 313 nurses and 316 physicians were interviewed. Among them, 1 midwife, 5 nurses and 27 physicians reported positions in which they were not in contact with patients (e.g., administration, management, expertise); thus, they were excluded from our analyses.

Among all healthcare professionals, 93.6% mentioned the availability of an HIV-testing service within their facility. However, only two-thirds mentioned the presence of a Voluntary HIV Testing Center (VTC) (Table 1). The presence of an ARV prescription service was mentioned by two-thirds of health professionals.

**Table 1:** Description of curricula, professional activity and professional structure among midwives, nurses and physicians, Côte d’Ivoire, DOD-CI study, 2018 (n = 895)

	Midwives (n = 298)		Nurses (n = 308)		Physicians (n = 289)	
	N	%	N	%	N	%
*Education and professional experience*						
Main activity						
Generalist	0	0.0	162	52.6	176	60.9
Specialist^a^	298	100.0	146	47.4	113	39.1
Years of professional practice						
≤ 3 years	211	70.8	184	59.7	74	25.6
≥ 4 years	87	29.2	124	40.3	215	74.4
Has already received specific training on HIV						
Yes	174	58.4	155	50.3	183	63.3
No	124	41.6	153	49.7	106	36.7
*Activities and services within the structure*						
Average number of patients cared for by the healthcare professional per day						
≤ 9	66	22.1	70	22.7	49	17.0
10–19	130	43.6	111	36.0	138	47.8
≥ 20	102	34.2	127	41.2	102	35.3
Type of health facility						
Hospital	141	47.3	175	56.8	146	50.5
Health center	143	48.0	111	36.0	47	16.3
Medical office or clinic	2	0.7	3	1.0	54	18.7
Other structure	12	4.0	19	6.2	42	14.5
Presence of a voluntary HIV-testing center						
Yes	193	65.6	178	62.9	180	69
No	89	30.3	93	32.9	69	26.4
Don’t know	12	4.1	12	4.2	12	4.6
Presence of community HIV counselors						
Yes	127	42.6	121	39.3	100	34.6
No	149	50.0	173	56.2	167	57.8
Don’t know	22	7.4	14	4.5	22	7.6
Presence of an ARV prescription service						
Yes	193	65.6	178	62.9	180	69.0
No	89	30.3	93	32.9	69	26.4
Don’t know	12	4.1	12	4.2	12	4.6
Satisfaction with available equipment and premises						
Very poorly/poorly equipped	147	49.3	174	56.5	117	40.4
Very good/well equipped	151	50.7	134	43.5	141	59.5
Opinion regarding confidentiality within the structure						
Confidentiality guaranteed	294	98.7	296	96.1	271	93.8
Confidentiality not guaranteed	4	1.3	9	2.9	16	5.5
Don’t know	0	0.0	3	1.0	2	0.7
Department						
Abidjan	83	27.9	70	22.7	183	63.3
Urban departments	58	19.5	68	22.1	37	12.8
Rural departments	157	52.7	170	55.2	69	23.9
*Opinions and perceptions*						
Belief that offering HIV testing systematically to any patient in medical consultation is						
Very useful	138	46.3	135	43.8	87	30.1
Useful	154	51.7	146	47.4	154	53.3
No opinion	3	1.0	10	3.2	6	2.1
Useless	3	1.0	17	5.5	42	14.5
Belief that HIV testing requires more caution in obtaining consent than other tests						
Yes	275	92.3	289	93.8	249	86.2
No	22	7.4	17	5.5	40	13.8
Don’t know	1	0.3	2	0.6	0	0.0
Knows an HIV-infected relative (excluding patients)						
Yes	159	53.4	209	67.9	223	77.2
No	139	46.6	99	32.1	66	22.8

^a^Specialized adult, prenatal, postnatal and family planning, pediatric, hospitalization and other consultations

Although almost all healthcare professionals were in contact with HIV-infected patients (96.4%), 53.3%, 67.8% and 77.1% of midwives, nurses and physicians, respectively, had a relative living with HIV (Table 1). Almost all healthcare professionals reported that HIV testing required specific consent compared to other diseases.

Nurses were the healthcare professional group who reported having been trained for HIV the least often (50.3% vs. 58.4% and 63.3% for midwives and physicians, respectively).

The median number of patients seen per day in consultation by health professionals was 15 (interquartile range 10–20).

### Number of Proposed Tests and Associated Factors

**Fig. 2:**
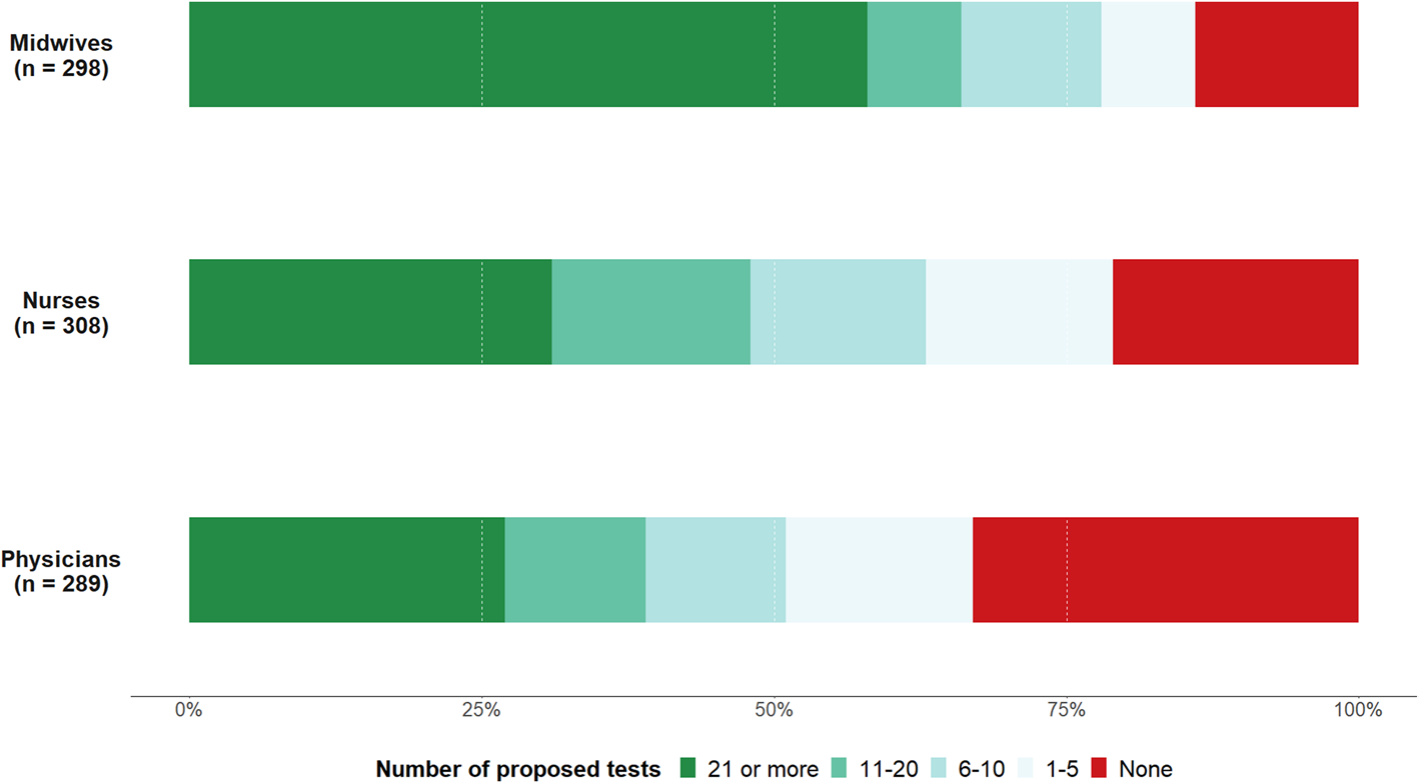
Number of proposed tests during the previous month among midwives, nurses and physicians, DOD-CI study, Côte d’Ivoire, 2018 (n = 895)

The number of proposed tests in the previous month differed by the medical profession (Fig. 2). Midwives reported a higher number of tests compared to nurses and physicians: 58.4% had proposed 21 tests or more in the previous month compared to 30.8% and 26.6% for nurses and physicians, respectively. The proportion of professionals who did not propose testing was higher among physicians (32.9% vs. 21.43% and 13.8% among nurses and midwives, respectively).

**Fig. 3:**
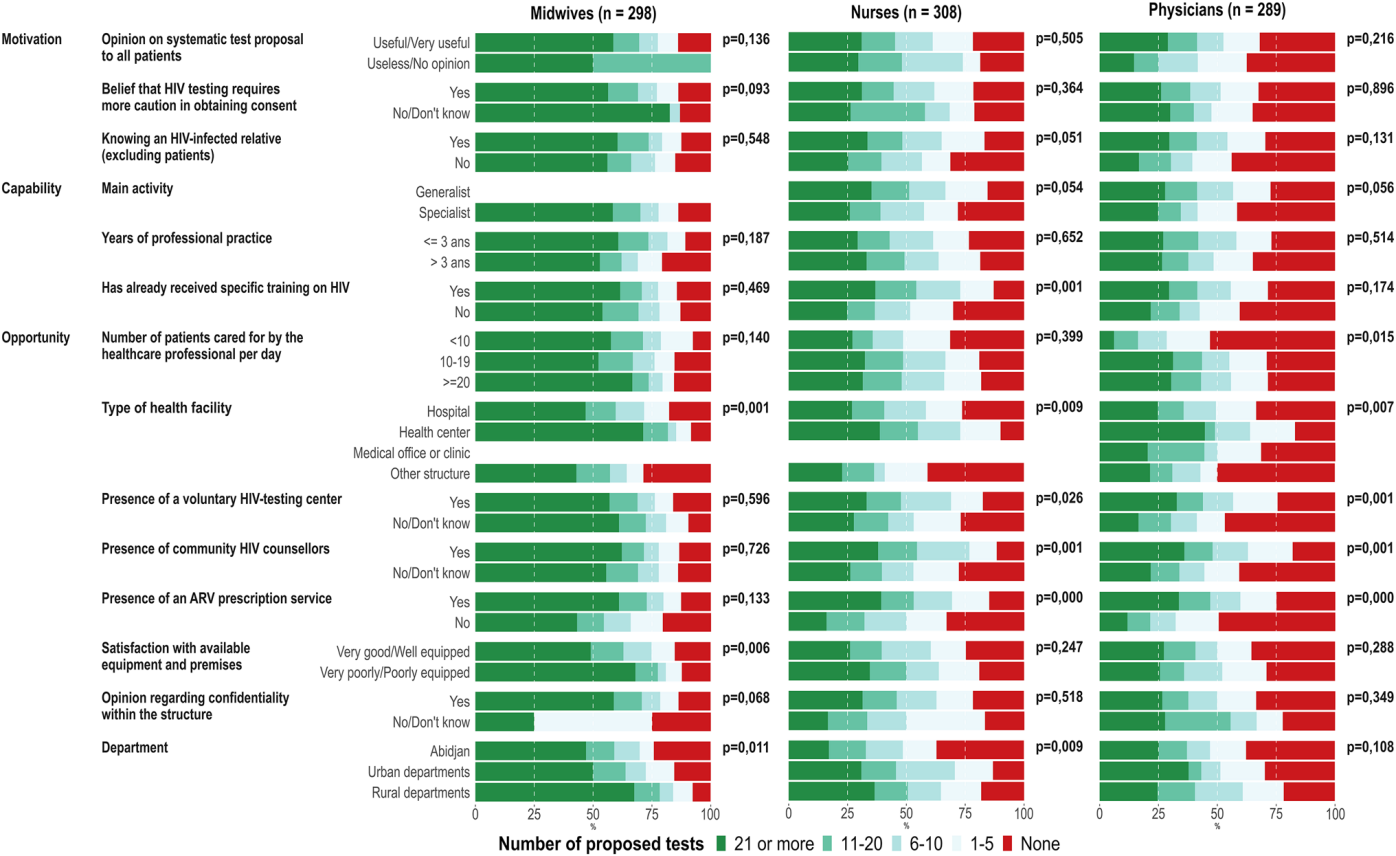
Factors associated with the number of proposed tests during the previous month among midwives, nurses and physicians, DOD-CI study, Côte d’Ivoire, 2018 (n = 895). Midwives and nurses working in a medical office or clinic are grouped with the other structures. p-values were computed with Fisher’s exact tests

In the bivariate analyses, there were different dimensions associated with the number of proposed tests according to professional position (Fig. 3; the corresponding data are presented in Table S2, SDC). Regarding motivation and capabilities, only knowing a relative living with HIV and having received HIV training were significantly associated with the number of tests proposed among nurses (exact F-test: p = 0.051 and p < 0.001 respectively). Regarding opportunities, the type of health facility was associated with the number of proposed tests regardless of the profession (the number of proposed tests was higher in health centers than in hospitals and other medical structures; exact F-test: p < 0.001, p = 0.009 and p = 0.007 among midwives, nurses and physicians respectively). The availability of a VTC, community HIV counselors and/or an ARV prescription service were significantly associated with a greater number of proposed tests for physicians and nurses.

**Fig. 4:**
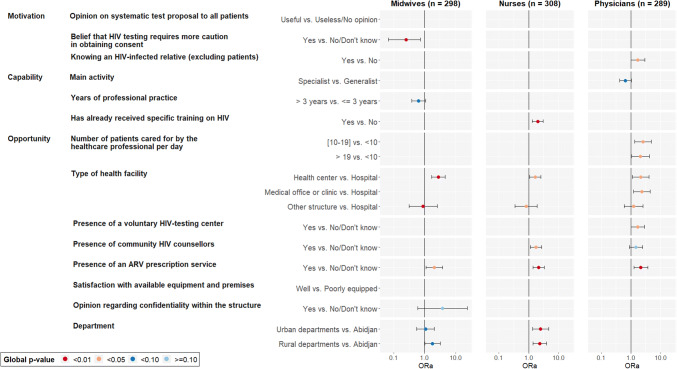
Ordinal regression model of factors associated with the number of proposed tests during the previous month depending on healthcare professionals’ motivations, capabilities and opportunities, by profession, Côte d’Ivoire, DOD-CI study, 2018 (n = 895). Midwives and nurses working in a medical office or clinic are grouped with the other structures. Likelihood ratio tests were used to calculate the global p-values

In the multivariate ordinal regression models, factors related to motivation (opinion on the collection of consent for HIV testing) and opportunities (type of health facility and presence of an ARV prescription service in this facility) were significantly associated with the number of proposed tests for midwives (Fig. 4). Among midwives, the belief that more caution is needed to obtain consent for HIV testing than for other diseases was associated with a lower number of proposed tests (aOR 0.25 [95% confidence interval 0.07–0.73], p = 0.010) (Table S3, SDC).

Among nurses, factors related to capabilities (having received specific training on HIV) and opportunities (type of health facility, presence of community HIV counselors, availability of an ARV prescription service and geographical location of the health facility outside Abidjan) were significantly associated with the number of proposed tests (Fig. 4). Having received specific training on HIV and the presence of community HIV counselors in the main facility were associated with a higher number of proposed tests (aOR 2.01 [1.31–3.09], p < 0.001 and aOR 1.75 [1.14–2.70], p = 0.011, respectively) (Table S3, SDC).

Among physicians, factors related to motivation (knowing a relative living with HIV) and opportunities (type of health facility, number of patients seen per day, presence of a VCT and an ARV prescription service in this facility) were significantly associated with the number of proposed tests (Fig. 4). Knowing a relative living with HIV and the presence of a VTC in the health facility were associated with a higher number of proposed tests (aOR 1.71 [1.01–2.94], p = 0.048, and aOR 1.69 [1.01–2.86], p = 0.047, respectively) (Table S3, SDC).

## Discussion

Our results showed different PITC practices depending on the medical profession. Midwives proposed HIV testing more often, followed by nurses and physicians. However, the number of proposed tests seemed relatively low compared to the number of patients seen in consultation. In fact, while healthcare professionals reported a median of 15 patients each day, only 39% reported 21 or more proposed tests during the previous month. The low number of proposed tests seems consistent with other studies conducted in West and Central Africa that show low rates of HIV testing proposal even during recommended events [27–30].The factors associated with PITC practice were also variable according to the medical profession. Environmental opportunities such as the presence of dedicated HIV testing services or dedicated staff increased the number of tests proposed by nurses and physicians. Among midwives, PITC practice was associated with their perception (thinking that HIV testing requires a specific consent procedure). Capability was not associated with PITC practice among physicians and midwives, while for nurses, specific HIV training was strongly associated with a higher number of proposed tests.

Among midwives, the high number of proposed tests suggests routine proposal resulting from their frequent contact with pregnant women for whom a systematic screening test is clearly recommended [31]. Indeed, due to the long history of prevention of mother-to-child transmission programs, midwives are more likely to be informed about this recommendation and to have learned it during their basic training. This could explain the lesser influence of their capabilities and of environmental opportunities on their PITC practice because PITC is an integral part of their current practice. PITC practice among midwives seemed to be related to their perceptions: the 8% of those who did not perceive the need to obtain specific consent for HIV testing were more likely to propose a higher number of tests. However, it is difficult to know whether the perception of specific consent influences midwives’ PITC practice or whether the practice of routine PITC changes midwives’ perceptions about obtaining consent. Other studies have noted difficulties in obtaining consent and therefore difficulty in proposing the test as barriers to PITC [8, 9, 12]. Simplifying consent collection and standardizing PITC in everyday medical practice should facilitate routine PITC practice.

Whereas PITC was seen as an integral part of midwives’ work, physicians and nurses perceived PITC as a ‘separate activity’ requiring dedicated staff, training and funding [22]. This could explain the lower number of proposed tests by nurses and physicians as well as the positive association between this number and specific HIV training (for nurses) and the presence of staff or places dedicated to HIV testing (for both nurses and physicians).

Additionally, the less frequent practice of PITC among nurses and physicians could result from unclear national recommendations in Côte d’Ivoire, which stipulate a routine testing proposal to any person seen in consultation while drawing a somewhat ‘broad’ prioritization for certain audiences [31]:

Since Côte d’Ivoire is a country with a mixed HIV epidemic, HIV testing services must be available to all people, regardless of age and socio-professional group. However, the availability of testing services will be increased in high-prevalence areas and for vulnerable populations most at risk of HIV infection. These vulnerable populations, priority targets for screening, are: symptomatic patients (suggestive signs of HIV infection, signs of malnutrition, signs of sexually transmitted infection, signs of tuberculosis), sex workers and their stable partners, men who have sex with men, truck drivers, prison populations, drug users including injectable drug users, men in uniform, migrants, truck drivers, orphans and vulnerable children, children born from HIV-positive mothers, pregnant women and their spouses, adolescents and young girls, persons aged 40 and over and family members of index subjects’

International donors such as PEPFAR (which funds 77% of the fight against HIV in Côte d’Ivoire) have a stronger targeting strategy [32]. These discrepancies between the strategies of political decision makers and donors could explain the lesser involvement of nurses and physicians in routine PITC. In practice, physicians and nurses carry out PITC in a targeted manner, mainly based on clinical criteria [22].

The nonsystematic and autonomous PITC practice among physicians and nurses may explain the greater number of factors associated with PITC practice compared to midwives. However, these factors differed between nurses and physicians.

Nurses’ capabilities, including having received HIV training, appeared to be related to the number of proposed tests, which was not the case for other professionals. Other data from our study showed that 20.8% of nurses reported difficulties in offering testing that were mainly related to the patient-caregiver relationship (e.g., reluctance to propose the test to certain audiences, fear of reporting a positive result, fear of having their testing proposal rejected; results not presented). These difficulties, which are related to counseling management, were also documented in other African contexts [8–12]. HIV-specific training would make it easier for nurses to address the issue of testing their patients, especially since only half of nurses have already received specific training related to HIV.

Among physicians, PITC practice was related to their motivation or perceptions more than to their capability. This difference could be explained by the greater autonomy of physicians in their professional practice. Physicians practice PITC when they are personally sensitized to it (e.g., by knowing a relative living with HIV), while nurses practice it when they are professionally sensitized (e.g., dedicated HIV training). A study conducted in Côte d’Ivoire in 2014 showed that physicians had a lack of interest and motivation in the practice of PITC, which underlines the importance of physicians’ motivations in their PITC practice [22].

The environmental opportunities, including the model of PITC organization, were linked to the practice of PITC among both nurses and physicians. The presence of community HIV counselors and/or a voluntary testing center seemed to increase the number of proposed tests. Healthcare professionals may be more inclined to offer HIV testing when it is not necessary for them to perform all the testing steps (i.e., counseling, blood sampling, and result announcement), which they may consider time-consuming or not part of their work [18, 22].

Some environmental opportunities linked to PITC practice were common to all professionals. The absence of an ARV prescription service was an obstacle to PITC for all healthcare professionals. The inability to deliver ARV treatment onsite following a positive result could create reluctance among healthcare professionals to initiate PITC.

In theory, the number of proposed tests should increase with the number of patients seen in consultation, although this was not the case for midwives and nurses. It is possible that managing a large number of patients, and therefore a higher workload, leaves less time to practice PITC. Studies tend to show that an overcrowded environment that involves many patients and long queues can force professionals to not offer the test due to a lack of time or insufficient staff [8–16].

Our study had some limitations, including memory bias that may have underestimated the number of proposed tests in consultation. However, the fact that the number of tests was limited to those proposed in the month preceding the survey may have reduced this bias. Reporting bias including desirability or uncoordinated responses between practice and reporting may have occurred since data were based on individual statement. To limit the impact of this effect, we have reassured participants that the study was anonymous and that no personal information was shared. In addition, we collected the number of proposed tests and the number of patients seen in consultation directly per interval at the time of collection; thus, we could not calculate test proposal rates (i.e., the ratio of the number of proposed tests to the number of patients seen in consultation).

Physician participation rates were lower than the rates for other health professionals but remained comparable to or higher than those in other studies conducted among physicians in this context [33, 34]. In addition, the distribution by sex and region of the different professionals in our sample was similar to the data in the annual report on the health situation in Côte d’Ivoire [35]. Our three samples of health professionals are therefore representative of their respective populations on a national scale.

The targeted practice of PITC among physicians and nurses raises the question of their ability to identify the most exposed audiences. Some populations are at risk because of their sexual behavior (e.g., multiple sexual partners, sex between men, commercial sex), yet their identification by healthcare professionals is often difficult because patients do not spontaneously communicate about their sexual practices, and health professionals do not routinely assess the risks of their patients [36, 37]. Given the trend toward targeted use of the PITC in both national policies and healthcare professional practice, clearly stated targeting modalities (e.g., the public concerned, modality for risk assessment for patients) seem necessary to avoid missing testing occasions for undiagnosed HIV patients.

## Conclusion

Although environment opportunities (such as dedicated staff or services) had an influence on PITC, capabilities and motivations had an effect of HIV testing proposals, but this effect differed by medical profession.

For midwives, routine integration of the HIV testing proposal as part of standard care appeared to be a key element for systematic HIV testing. However, there are remaining gaps, and the consent process could be simplified and aligned with other screening processes.

For nurses, improving their capabilities, particularly dedicated HIV testing training, can lead to better testing.

For physicians, motivations and perceptions are linked to their PITC practice, suggesting the need for actions to raise their awareness of PITC and clearer recommendations on when to test.

## Electronic supplementary material

Below is the link to the electronic supplementary material.

Supplementary file1 (DOCX 63 kb)

## Data Availability

*Data protection, confidentiality and privacy* Data has been anonymized; All identifying data (e.g. telephone numbers) have been deleted.
