# Supplementary material for: Practices and Obstacles to Provider-Initiated HIV Testing and Counseling (PITC) Among Healthcare Providers in Côte d’Ivoire
Source: AIDS Behav. 2020 May 24;24(12):3491–500. doi: 10.1007/s10461-020-02923-0 (PMC7667140; doi:10.1007/s10461-020-02923-0)
Supplement: Supplementary file 1 — Supplementary file1 (DOCX 63 kb) [file 10461_2020_2923_MOESM1_ESM.docx]

Table S1: Results of telephone calls from the surveys among midwives, nurses and physicians, DOD-CI study, Côte d’Ivoire, 2018.

|  |  | **Midwives** | | | |  | **Nurses** | |  | **Physicians** | |
| --- | --- | --- | --- | --- | --- | --- | --- | --- | --- | --- | --- |
|  |  | n | | | % |  | n | % |  | n | % |
|  | - | - | | | - |  | - | - |  | - | - |
|  | **Base of phone numbers used** | **425** | | | **100.0** |  | **425** | **100.0** |  | **600** | **100.0** |
| EF | Unable to be reached without any contact | 10 | | | 2.4 |  | 7 | 1.6 |  | 22 | 3.7 |
| OT | Respondent is not a healthcare professional | 17 | | | 4.0 |  | 14 | 3.3 |  | 28 | 4.7 |
| OT | Language barrier with the respondent | | 1 | | 0.2 |  | 1 | 0.2 |  | 4 | 0.7 |
| OT | Other healthcare professional | 1 | | | 0.2 |  | 15 | 3.5 |  | 2 | 0.3 |
|  | **Interviewable individuals** | **396** | | | **93.2** |  | **389** | **91.5** |  | **544** | **90.7** |
|  | - | - | | | - |  | - | - |  | - | - |
|  | **Interviewable individuals** | **396** | | | **100.0** |  | **389** | **100.0** |  | **544** | **100.0** |
| EF | Cannot be reached after appointment or line break, questionnaire not started | 73 | | | 18,4 |  | 41 | 10,5 |  | 144 | 26,5 |
| R | Refusal to participate in the survey | 23 | | | 5,8 |  | 24 | 6,2 |  | 83 | 15,3 |
|  | **Individuals interviewed** | **300** | | | **75.8** |  | **324** | **83.3** |  | **317** | **58.3** |
|  | - | - | | | - |  | - | - |  | - | - |
|  | **Individuals interviewed** | **300** | | | **100.0** |  | **324** | **100.0** |  | **317** | **100.0** |
| EE | Abandons | 1 | | | 0.3 |  | 11 | 3.4 |  | 1 | 0.3 |
|  | **Completed questionnaires** | **299** | | | **99.7** |  | **313** | **96.6** |  | **316** | **99.7** |
|  | - |  | | |  |  |  |  |  |  |  |
|  | **Overall refusal rate** (Eligible individuals with exclusion of off-target persons) | | | - | 5.8 |  | - | 6.2 |  | - | 15.3 |
|  | **Overall execution failure rate** (Eligible individuals with exclusion of off-target persons) | | | - | 20.7 |  | - | 14.9 |  | - | 29.5 |

EF: Execution Failures; OT: Out of Target; R: Refusal

**Table S2 (1/2): Factors Associated with the number of proposed tests during the previous month according to healthcare professionals’ motivation, capability and opportunity, by profession, DOD-CI study, Côte d’Ivoire, 2018 (n= 895).**

|  |  | **Midwives (n=298)** | | | | |  | | **Nurses (n=308)** | | | | | |  | | **Physicians (n=289)** | | | | | |  | |  |
| --- | --- | --- | --- | --- | --- | --- | --- | --- | --- | --- | --- | --- | --- | --- | --- | --- | --- | --- | --- | --- | --- | --- | --- | --- | --- |
|  |  | None | [1–5] | [6–10] | [11–20] | >20 | | p-value | | None | [1–5] | [6–10] | [11–20] | >20 | | p-value | | None | [1–5] | [6–10] | [11–20] | >20 | | P-value | |
| **Motivation** | |  |  |  |  |  | |  | |  |  |  |  |  | |  | |  |  |  |  |  | |  | |
|  | **Belief that offering HIV testing systematically to any patient in medical consultation is** | | |  |  |  | | **0.138** | |  |  |  |  |  | | **0.511** | |  |  |  |  |  | | **0.210** | |
|  | Useful or very useful | 14.0 | 8.6 | 7.9 | 11.0 | 58.6 | |  | | 21.7 | 17.1 | 16.0 | 14.2 | 31.0 | |  | | 32.0 | 15.4 | 11.2 | 12.4 | 29.0 | |  | |
|  | Useless/no opinion | 0.0 | 0.0 | 0.0 | 50.0 | 50.0 | |  | | 18.5 | 7.4 | 25.9 | 18.5 | 29.6 | |  | | 37.5 | 20.8 | 16.7 | 10.4 | 14.6 | |  | |
|  | **Belief that HIV testing requires more caution in obtaining consent than other tests** | | |  |  |  | | **0.103** | |  |  |  |  |  | | **0.373** | |  |  |  |  |  | | **0.893** | |
|  | Yes | 13.8 | 9.1 | 8.0 | 12.7 | 56.4 | |  | | 21.5 | 16.6 | 17.3 | 13.5 | 31.1 | |  | | 32.5 | 16.1 | 12.9 | 12.4 | 26.1 | |  | |
|  | No/Don’t know | 13.0 | 0.0 | 4.3 | 0.0 | 82.6 | |  | | 21.1 | 10.5 | 10.5 | 31.6 | 26.3 | |  | | 35.0 | 17.5 | 7.5 | 10.0 | 30.0 | |  | |
|  | **Knowing an HIV-infected relative (excluding patients)** | |  |  |  |  | | **0.546** | |  |  |  |  |  | | **0.055** | |  |  |  |  |  | | **0.121** | |
|  | Yes | 12.6 | 8.2 | 5.7 | 13.2 | 60.4 | |  | | 16.7 | 18.2 | 16.7 | 14.8 | 33.5 | |  | | 29.6 | 16.1 | 13.0 | 11.7 | 29.6 | |  | |
|  | No/Don’t know | 15.1 | 8.6 | 10.1 | 10.1 | 56.1 | |  | | 31.3 | 12.1 | 17.2 | 14.1 | 25.3 | |  | | 43.9 | 16.7 | 9.1 | 13.6 | 16.7 | |  | |
| **Capability** | |  |  |  |  |  | |  | |  |  |  |  |  | |  | |  |  |  |  |  | |  | |
|  | **Main activity** |  |  |  |  |  | | **-** | |  |  |  |  |  | | **0.059** | |  |  |  |  |  | | **0.055** | |
|  | Generalist | - | - | - | - | - | |  | | 15.4 | 17.9 | 15.4 | 16.0 | 35.2 | |  | | 27.3 | 15.9 | 15.3 | 13.6 | 27.8 | |  | |
|  | Specialist | 13.8 | 8.4 | 7.7 | 11.7 | 58.4 | |  | | 28.1 | 14.4 | 18.5 | 13.0 | 26.0 | |  | | 41.6 | 16.8 | 7.1 | 9.7 | 24.8 | |  | |
|  | **Years of professional practice** | |  |  |  |  | | **0.187** | |  |  |  |  |  | | **0.659** | |  |  |  |  |  | | **0.519** | |
|  | ≤ 3 years | 10.9 | 7.6 | 8.1 | 12.8 | 60.7 | |  | | 23.4 | 15.2 | 18.5 | 13.6 | 29.3 | |  | | 27.0 | 14.9 | 16.2 | 14.9 | 27.0 | |  | |
|  | > 3 years | 20.7 | 10.3 | 6.9 | 9.2 | 52.9 | |  | | 18.5 | 17.7 | 14.5 | 16.1 | 33.1 | |  | | 34.9 | 16.7 | 10.7 | 11.2 | 26.5 | |  | |
|  | **Has already received specific training on HIV** | | |  |  |  | | **0.475** | |  |  |  |  |  | | **0.003** | |  |  |  |  |  | | **0.177** | |
|  | Yes | 14.4 | 8.0 | 6.9 | 9.2 | 61.5 | |  | | 12.9 | 14.2 | 18.7 | 17.4 | 36.8 | |  | | 28.4 | 15.8 | 14.2 | 12.0 | 29.5 | |  | |
|  | No/Don’t know | 12.9 | 8.9 | 8.9 | 15.3 | 54.0 | |  | | 30.1 | 18.3 | 15.0 | 11.8 | 24.8 | |  | | 40.6 | 17.0 | 8.5 | 12.3 | 21.7 | |  | |

**Table S2 (2/2): Factors associated with the number of proposed tests during the previous month according to healthcare professionals’ motivation, capability and opportunity, by profession, DOD-CI study, Côte d’Ivoire, 2018 (n= 895).**

|  |  | **Midwives (n=298)** | | | | |  | | **Nurses (n=308)** | | | | | | |  | | **Physicians (n=289)** | | | | | |  | |  |
| --- | --- | --- | --- | --- | --- | --- | --- | --- | --- | --- | --- | --- | --- | --- | --- | --- | --- | --- | --- | --- | --- | --- | --- | --- | --- | --- |
|  |  | None | [1–5] | [6–10] | [11–20] | >20 | | p-value | | None | [1–5] | [6–10] | [11–20] | >20 | | | p-value | | None | [1–5] | [6–10] | [11–20] | >20 | | p-value | |
| **Opportunity** | |  |  |  |  |  | |  | |  |  |  |  |  | | |  | |  |  |  |  |  | |  | |
|  | **Average number of patients cared for by the healthcare professional per day** | | |  |  |  | | **0.141** | |  |  |  |  |  | | | **0.391** | |  |  |  |  |  | | **0.014** | |
|  | <10 | 7.6 | 13.6 | 7.6 | 13.6 | 57.6 | |  | | 31.4 | 20.0 | 12.9 | 8.6 | 27.1 | | |  | | 53.1 | 18.4 | 12.2 | 10.2 | 6.1 | |  | |
|  | [10–19] | 15.4 | 8.5 | 9.2 | 14.6 | 52.3 | |  | | 18.9 | 14.4 | 18.0 | 16.2 | 32.4 | | |  | | 29.0 | 15.9 | 11.6 | 12.3 | 31.2 | |  | |
|  | > 19 | 15.7 | 4.9 | 5.9 | 6.9 | 66.7 | |  | | 18.1 | 15.7 | 18.1 | 16.5 | 31.5 | | |  | | 28.4 | 15.7 | 12.7 | 12.7 | 30.4 | |  | |
|  | **Type of health facility** | |  |  |  |  | | **0.001** | |  |  |  |  |  | | | **0.010** | |  |  |  |  |  | | **0.005** | |
|  | Hospital | 17.7 | 10.6 | 12.1 | 12.8 | 46.8 | |  | | 26.3 | 15.4 | 17.7 | 13.7 | 26.9 | | |  | | 33.6 | 17.1 | 13.7 | 11.0 | 24.7 | |  | |
|  | Health center | 8.4 | 6.3 | 3.5 | 10.5 | 71.3 | |  | | 9.9 | 17.1 | 18.0 | 16.2 | 38.7 | | |  | | 17.0 | 19.1 | 14.9 | 4.3 | 44.7 | |  | |
|  | Medical office or clinic | - | - | - | - | - | |  | | - | - | - | - | - | | |  | | 31.5 | 18.5 | 5.6 | 24.1 | 20.4 | |  | |
|  | Other facility | 28.6 | 7.1 | 7.1 | 14.3 | 42.9 | |  | | 40.9 | 18.2 | 4.5 | 13.6 | 22.7 | | |  | | 50.0 | 7.1 | 11.9 | 9.5 | 21.4 | |  | |
|  | **Presence of a VTC** |  |  |  |  |  | | **0.599** | |  |  |  |  |  | | | **0.028** | |  |  |  |  |  | | **0.001** | |
|  | Yes | 16.1 | 7.8 | 7.3 | 11.9 | 57.0 | |  | | 17.4 | 13.5 | 21.3 | 14.6 | 33.1 | | |  | | 24.4 | 18.9 | 12.8 | 11.1 | 32.8 | |  | |
|  | No/Don’t know | 9.5 | 9.5 | 8.6 | 11.4 | 61.0 | |  | | 26.9 | 20.0 | 10.8 | 14.6 | 27.7 | | |  | | 46.8 | 11.9 | 11.0 | 13.8 | 16.5 | |  | |
|  | **Presence of community HIV counselors** | | |  |  |  | | **0.726** | |  |  |  |  |  | | | **0.001** | |  |  |  |  |  | | **0.001** | |
|  | Yes | 13.4 | 8.7 | 6.3 | 9.4 | 62.2 | |  | | 11.6 | 11.6 | 22.3 | 16.5 | 38.0 | | |  | | 18.0 | 19.0 | 15.0 | 12.0 | 36.0 | |  | |
|  | No/Don’t know | 14.0 | 8.2 | 8.8 | 13.5 | 55.6 | |  | | 27.8 | 19.3 | 13.4 | 13.4 | 26.2 | | |  | | 40.7 | 14.8 | 10.6 | 12.2 | 21.7 | |  | |
|  | **Presence of an ARV prescription service** | | |  |  |  | | **0.132** | |  |  |  |  |  | **<0.001** | | | |  |  |  |  |  | | **<0.001** | |
|  | Yes | 12.6 | 7.5 | 7.1 | 11.8 | 61.0 | |  | | 14.8 | 15.8 | 16.3 | 13.8 | 39.3 | | |  | | 25.0 | 15.3 | 12.8 | 13.3 | 33.7 | |  | |
|  | No/Don’t know | 20.5 | 13.6 | 11.4 | 11.4 | 43.2 | |  | | 33.0 | 17.0 | 17.9 | 16.1 | 16.1 | | |  | | 49.5 | 18.3 | 10.8 | 9.7 | 11.8 | |  | |
|  | **Satisfaction with available equipment and premises** | |  |  |  |  | | **0.005** | |  |  |  |  |  | | | **0.236** | |  |  |  |  |  | | **0.288** | |
|  | Well equipped | 15.2 | 9.9 | 11.9 | 13.9 | 49.0 | |  | | 24.6 | 14.9 | 20.9 | 13.4 | 26.1 | | |  | | 35.5 | 14.5 | 9.3 | 13.4 | 27.3 | |  | |
|  | Poorly equipped | 12.2 | 6.8 | 3.4 | 9.5 | 68.0 | |  | | 19.0 | 17.2 | 13.8 | 15.5 | 34.5 | | |  | | 29.1 | 18.8 | 16.2 | 10.3 | 25.6 | |  | |
|  | **Opinion regarding confidentiality within the structure** | |  |  |  |  | | **0.067** | |  |  |  |  |  | | | **0.534** | |  |  |  |  |  | | **0.351** | |
|  | Guaranteed | 13.6 | 7.8 | 7.8 | 11.9 | 58.8 | |  | | 21.6 | 15.5 | 16.9 | 14.5 | 31.4 | | |  | | 33.6 | 16.6 | 12.2 | 11.1 | 26.6 | |  | |
|  | Not guaranteed/ Don’t know | 25.0 | 50.0 | 0.0 | 0.0 | 25.0 | |  | | 16.7 | 33.3 | 16.7 | 16.7 | 16.7 | | |  | | 22.2 | 11.1 | 11.1 | 27.8 | 27.8 | |  | |
|  | **Department** |  |  |  |  |  | | **0.012** | |  |  |  |  |  | | | **0.011** | |  |  |  |  |  | | **0.104** | |
|  | Abidjan | 24.1 | 6.0 | 10.8 | 12.0 | 47.0 | |  | | 37.1 | 14.3 | 15.7 | 15.7 | 17.1 | | |  | | 37.7 | 15.3 | 9.8 | 12.0 | 25.1 | |  | |
|  | Urban departments | 15.5 | 12.1 | 8.6 | 13.8 | 50.0 | |  | | 13.2 | 16.2 | 25.0 | 14.7 | 30.9 | | |  | | 29.7 | 18.9 | 8.1 | 5.4 | 37.8 | |  | |
|  | Rural departments | 7.6 | 8.3 | 5.7 | 10.8 | 67.5 | |  | | 18.2 | 17.1 | 14.1 | 14.1 | 36.5 | | |  | | 21.7 | 17.4 | 20.3 | 15.9 | 24.6 | |  | |

ARV: Antiretroviral; VTC: Voluntary HIV-Testing Center

note 1: Midwives and nurses working in a medical office or clinic are grouped with the other structures.

note 2: Fisher’s exact tests were used to calculate the p-values

**Table S3 (1/2): Multivariate ordinal model of factors associated with the number of proposed tests during the previous month among midwives, nurses and doctors, in Côte d’Ivoire, DOD-CI study, 2018 (n=895).**

|  |  | **Midwives (n=298)** | | |  | **Nurses (n=308)** | | |  | **Physicians (n=289)** | | |
| --- | --- | --- | --- | --- | --- | --- | --- | --- | --- | --- | --- | --- |
|  |  | **aOR** | **IC95%** | **p-value** |  | **aOR** | **IC95%** | **p-value** |  | **aOR** | **IC95%** | **p-value** |
| **Motivation** | **Belief that offering HIV testing systematically to any patient in medical consultation is** |  |  |  |  |  |  |  |  |  |  |  |
|  | Useful or very useful | - | - |  |  | - | - |  |  | - | - |  |
|  | Useless/no opinion | - | - |  |  | - | - |  |  | - | - |  |
|  | **Belief that HIV testing requires more caution in obtaining consent than other tests** |  |  | **0.010** |  |  |  |  |  |  |  |  |
|  | Yes | 0.25 | 0.07-0.73 |  |  | - | - |  |  | - | - |  |
|  | No/Don’t know | *ref.* | *ref.* |  |  | - | - |  |  | - | - |  |
|  | **Knowing an HIV-infected relative (excluding patients)** |  |  |  |  |  |  |  |  |  |  | **0.048** |
|  | Yes | - | - |  |  | - | - |  |  | 1.71 | 1.01–2.94 |  |
|  | No/Don’t know | - | - |  |  | - | - |  |  | *ref.* | *ref.* |  |
| **Capability** | **Main activity** |  |  |  |  |  |  |  |  |  |  | **0.064** |
|  | Generalist | - | - |  |  | - | - |  |  | *ref.* | *ref.* |  |
|  | Specialist | - | - |  |  | - | - |  |  | 0.65 | 0.42–1.02 |  |
|  | **Years of professional practice** |  |  | **0.094** |  |  |  |  |  |  |  |  |
|  | ≤ 3 years | *ref.* | *ref.* |  |  | - | - |  |  | - | - |  |
|  | > 3 years | 0.64 | 0.38–1.08 |  |  | - | - |  |  | - | - |  |
|  | **Has already received specific training on HIV** |  |  |  |  |  |  | **0.001** |  |  |  |  |
|  | Yes | - | - |  |  | 2.01 | 1.31–3.09 |  |  | - | - |  |
|  | No/Don’t know | - | - |  |  | *ref.* | *ref.* |  |  | - | - |  |
| **Opportunity** | **Average number of patients cared for by the healthcare professional per day** |  |  |  |  |  |  |  |  |  |  | **0.016** |
|  | <10 | - | - |  |  | - | - |  |  | *ref.* | *ref.* |  |
|  | [10–19] | - | - |  |  | - | - |  |  | 2.53 | 1.34–4.86 |  |
|  | > 19 | - | - |  |  | - | - |  |  | 2.09 | 1.06–4.17 |  |
|  | **Type of facility** |  |  | **< 0.001** |  |  |  | **0.042** |  |  |  | **0.016** |
|  | Hospital | *ref.* | *ref.* |  |  | *ref.* | *ref.* |  |  | *ref.* | *ref.* |  |
|  | Health center | 2.76 | 1.68–4.57 |  |  | 1.68 | 1.09- 2.60 |  |  | 2.13 | 1.13–4.08 |  |
|  | Medical office or clinic | - | - |  |  | - | - |  |  | 2.32 | 1.24–4.39 |  |
|  | Other facility | 0.90 | 0.32–2.60 |  |  | 0.82 | 0.34- 1.92 |  |  | 1.24 | 0.59–2.58 |  |

**Table S3 (2/2): Multivariate ordinal model of factors associated with the number of proposed tests during the previous month among midwives, nurses and doctors, in Côte d’Ivoire, DOD-CI study, 2018 (n=895).**

|  |  | **Midwives (n=298)** | | |  | **Nurses (n=308)** | | |  | **Physicians (n=289)** | | |
| --- | --- | --- | --- | --- | --- | --- | --- | --- | --- | --- | --- | --- |
|  |  | **aOR** | **IC95%** | **p-value** |  | **aOR** | **IC95%** | **p-value** |  | **aOR** | **IC95%** | **p-value** |
| **Opportunity** | **Presence of a voluntary HIV-testing center** |  |  |  |  |  |  |  |  |  |  | **0.047** |
|  | Yes | - | - |  |  | - | - |  |  | 1.69 | 1.01–2.86 |  |
|  | No/Don’t know | - | - |  |  | - | - |  |  | *ref.* | *ref.* |  |
|  | **Presence of community HIV counselors** |  |  |  |  |  |  | **0.011** |  |  |  | **0.134** |
|  | Yes | - | - |  |  | 1.75 | 1.14–2.70 |  |  | 1.49 | 0.88–2.50 |  |
|  | No/Don’t know | - | - |  |  | *ref.* | *ref.* |  |  | *ref.* | *ref.* |  |
|  | **Presence of an ARV prescription service** |  |  | **0.023** |  |  |  | **<0.001** |  |  |  | **0.004** |
|  | Yes | 2.07 | 1.11–3.83 |  |  | 2.15 | 1.40–3.31 |  |  | 2.16 | 1.28–3.68 |  |
|  | No/Don’t know | *ref.* | *ref.* |  |  | *ref.* | *ref.* |  |  | *ref.* | *ref.* |  |
|  | **Satisfaction with available equipment and premises** |  |  |  |  |  |  |  |  |  |  |  |
|  | Well equipped | - | - |  |  | - | - |  |  | - | - |  |
|  | Poorly equipped | - | - |  |  | - | - |  |  | - | - |  |
|  | **Opinion regarding confidentiality within the structure** |  |  | **0.145** |  |  |  |  |  |  |  |  |
|  | Guaranteed | 3.84 | 0.61-24.15 |  |  | - | - |  |  | - | - |  |
|  | Not guaranteed/Don’t know | *ref.* | *ref.* |  |  | - | - |  |  | - | - |  |
|  | **Department** |  |  | **0.072** |  |  |  | **0.002** |  |  |  |  |
|  | Abidjan | *ref.* | *ref.* |  |  | *ref.* | *ref.* |  |  | - | - |  |
|  | Urban departments | 1.08 | 0.56-2.08 |  |  | 2.52 | 1.37-4.66 |  |  | - | - |  |
|  | Rural departments | 1.81 | 1.02-3.20 |  |  | 2.36 | 1.41-3.97 |  |  | - | - |  |

ARV: Antiretroviral; VTC: Voluntary HIV-Testing Center

note 1: Midwives and nurses working in a medical office or clinic are grouped with the other structures.

note 2: likelihood ratio tests were used to calculate the global p-values
